# Positive Feedback between Mycorrhizal Fungi and Plants Influences Plant Invasion Success and Resistance to Invasion

**DOI:** 10.1371/journal.pone.0012380

**Published:** 2010-08-24

**Authors:** Qian Zhang, Ruyi Yang, Jianjun Tang, Haishui Yang, Shuijin Hu, Xin Chen

**Affiliations:** 1 College of Life Sciences, Zhejiang University, Hangzhou, China; 2 College of Environmental Science, Anhui Normal University, Wuhu, China; 3 Department of Plant Pathology, North Carolina State University, Raleigh, North Carolina, United States of America; Agroscope Reckenholz-Tänikon, Research Station ART, Switzerland

## Abstract

Negative or positive feedback between arbuscular mycorrhizal fungi (AMF) and host plants can contribute to plant species interactions, but how this feedback affects plant invasion or resistance to invasion is not well known. Here we tested how alterations in AMF community induced by an invasive plant species generate feedback to the invasive plant itself and affect subsequent interactions between the invasive species and its native neighbors. We first examined the effects of the invasive forb *Solidago canadensis* L. on AMF communities comprising five different AMF species. We then examined the effects of the altered AMF community on mutualisms formed with the native legume forb species *Kummerowia striata* (Thunb.) Schindl. and on the interaction between the invasive and native plants. The host preferences of the five AMF were also assessed to test whether the AMF form preferred mutualistic relations with the invasive and/or the native species. We found that *S. canadensis* altered AMF spore composition by increasing one AMF species (*Glomus geosporum*) while reducing *Glomus mosseae*, which is the dominant species in the field. The host preference test showed that *S. canadensis* had promoted the abundance of AMF species (*G. geosporum*) that most promoted its own growth. As a consequence, the altered AMF community enhanced the competitiveness of invasive *S. canadensis* at the expense of *K. striata*. Our results demonstrate that the invasive *S. canadensis* alters soil AMF community composition because of fungal-host preference. This change in the composition of the AMF community generates positive feedback to the invasive *S. canadensis* itself and decreases AM associations with native *K. striata*, thereby making the native *K. striata* less dominant.

## Introduction

Because of their ubiquity and presumed low level of host specificity, arbuscular mycorrhizal fungi (AMF) have been generally believed to play a minor role in mediating the invasion of exotic plants [1]. However, increasing evidence indicates that specific host-fungal pairings exist [2], [3]. Some AMF species are more beneficial to a host plant than are others [2]–[7], and certain AMF are differentially promoted by different plant hosts [8], [9] due to preferential allocation of photosynthate by host plants [10]. Experiments also demonstrated that the identity of AMF species can impact the performance of invasive plants [11], [12]. This evidence of specific host-fungal interactions suggests that AMF could affect plant invasion.

Host-fungal specificity can lead to different AMF communities in roots of co-occurring plant species [13], [14] and has also been presumed to enable invasive plant species to alter the density and composition of the indigenous AMF community [15], [16]. When exposed to a mixture of indigenous AMF species, invasive plants were colonized by one or more AMF species that differed from those that colonized the tested native hosts [15], [17]. Therefore, invasive species could be more successful in the presence of certain AMF species, and this could increase the abundance of those AMF species [11] and possibly change the AMF community composition. This assumption, however, has rarely been experimentally tested.

A shift in the AMF community driven by invasive plants may impact invasive and native plants differently because, as noted earlier, AMF do vary in host preference [2], [3]. Thus, predicting how the shifted AMF communities will affect the outcome of competition between invasive and native plants may depend on understanding the positive feedback between specific AMF and specific invasive hosts [18], [19]. When an invading species encounters and develops strong mutualisms with specific AMF, benefits to the AMF may generate positive feedback that enhances the persistence and abundance of the invasive host [20], helping the invasive host to compete with native plants. On the other hand, if the invaders are less responsive to the AMF species or are not mycorrhizal hosts at all, populations of AMF fungi could decline as plant invasion proceeds [16]. This decline could then reduce the formation of native plant mutualisms and thereby reduce the growth and competitive ability of native hosts, again resulting in positive feedback to invasive hosts.

Although there is a great deal of evidence demonstrating changes in AMF communities during exotic plant invasion [15], [21]–[23], the mechanisms underlying these changes and the consequences of these changes have not been well elucidated. Here we wanted to test how invasive plants alter indigenous arbuscular mycorrhizal fungal (AMF) communities and how the changed AMF communities affect invasive plants in their competition with native plants. We hypothesize that invasive plants may alter indigenous AMF communities by establishing preferred mutualisms that favor the invasive plant itself (positive feedback). These new mutualisms with invasive plants may lead to the decline in mutualisms with native plants and may favor invasive plants in their competition with native plants. We tested these hypotheses in greenhouse experiments by using the invasive forb *Solidago canadensis* L. and the native legume forb *Kummerowia striata* (Thunb.) Schindl.

*Solidago canadensis* L. (goldenrod) is a successful worldwide invader of North American origin [24] that has established in Europe, large parts of Asia, Australia, and New Zealand [25], [26]. This invasive weed spreads rapidly in southeastern China, invading abandoned fields and disturbed habitats [26]. In a previous three-year field study, we compared the AMF associated with several native species in the presence or absence of the invasive *S. canadensis* by analyzing soil and root samples from field sites. We found that *S. candensis* reduced AMF colonization of some native plants (*K. striata*, *Lolium perenne*, *Echinochloa crusgalli*, and *Ageratum conyzoides*) and altered AMF spore composition [27], [28]. Richness and abundance of AMF species that colonized the roots of the native *K. striata* differed in fields dominated by *S. canadensis* than in fields without *S. canadensis* [28].

In the present study, we first created an AMF community with five species that commonly exist in the field and examined the divergence of the constructed AMF community when pots were planted with the invasive *S. canadensis* or with the native *K. striata* (experiment 1). Changes in AMF community composition were assessed using spore counts (experiment 1) and with molecular tools (experiment 2). To measure feedback, we then examined the effects of the altered AMF community on the mutualisms formed with native *K. striata* and on the interaction between invasive and native plants (experiment 3). The host preference of the five AMF was also assessed to test whether the AMF form preferred mutualistic relations with the invasive species (experiment 4). All experiments were performed in the greenhouse with plants and AMF that coexist in the field.

## Results

### Effects of a native and an exotic plant on AMF community composition (experiment 1)

**Figure 1:**
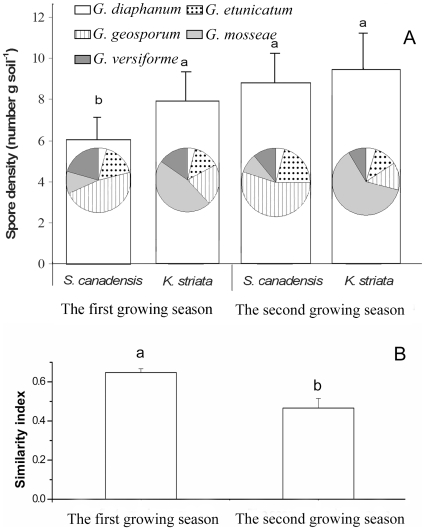
Numbers of AMF spores (total and by AMF species) in soil grown with invasive *S. canadensis* or native *K. striata* in two growing years in experiment 1. Bars represent total spore numbers of the five AMF. Inset pie charts represent spore composition of the AMF species. Values are means ± standard error. Means with different letters are significantly different at the 5% level.

The spore composition of the AMF community differed under the two hosts (Fig. 1): after the two growing seasons in experiment 1, *Glomus geosporum* spores were dominant under the invasive host (F_1,14_ = 37.64, P = 0.000) while *Glomus mosseae* spores were dominant under the native host (F_1,14_ = 89.71, P = 0.000) (Fig. 1A). There was no significant change in spore numbers of *Glomus versiforme* (F_1,14_ = 1.61, P = 0.225), but significantly different spore numbers of *Glomus diaphanum* (F_1,14_ = 5.28, P = 0.038) and *Glomus etunicatum* (F_1, 14_ = 9.29, P = 0.009) were found under the two host plants after the two growing season (Fig. 1A). The total numbers of AMF spores (F_1,14_ = 4.04, P = 0.064, Fig. 1A) were not different, but AMF communities diverged (in terms of Bray-Curtis similarity decreased, F_1,6_ = 77.15, P = 0.000, Fig. 1B) between the two host plants after the two growing seasons.

### Changes in the abundance of *G. mosseae* in native roots based on DNA (experiment 2)

**Figure 2:**
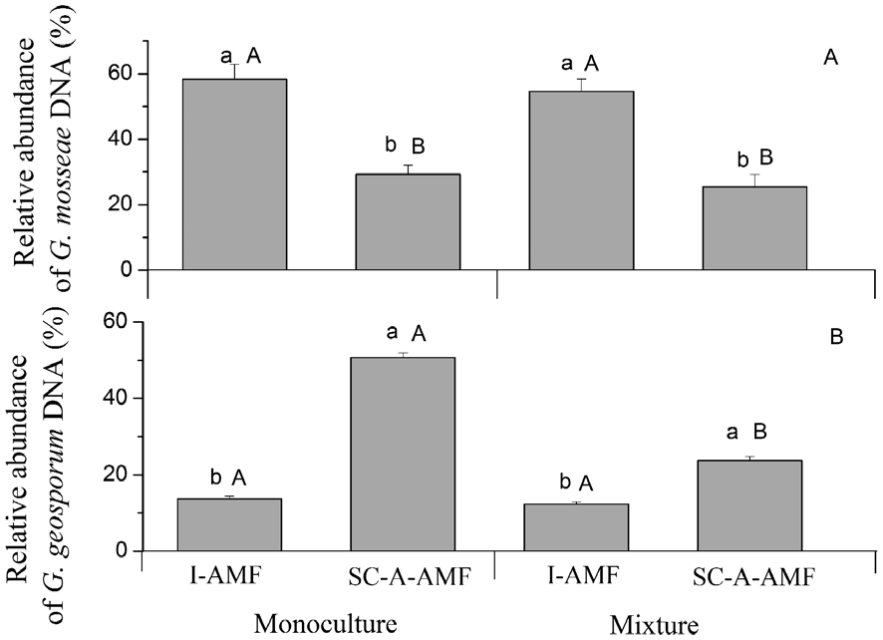
The relative abundance of DNA of *G. mosseae* (A) or *G. geosporum* (B) in roots of *K. striata* grown in soil containing the initial AMF community (I-AMF) or the AMF community altered by the invasive *S. canadensis* (SC-A-AMF) under monoculture and mixed planting with *S. canadensis* in experiment 2. The relative abundance of DNA of *G. mosseae*, or *G. geosporum* (%),  = I_g_/I_t_,×100, where I_g_ is the intensity of the *G. mosseae* band or *G. geosporum* band, and I_t_ is the total intensity of all the AMF species bands in one profile. Values are means ± standard error. Within monoculture or within mixed plantings, means with different lower case letters are significantly different at the 5% level. Within each AMF treatment (I-AMF or SC-A-AMF), means with different capital letters are significantly different at the 5% level.

According to the nested PCR-DGGE-sequencing method in experiment 2, the relative abundances of DNA *G. mosseae* and *G. geosporum* in roots of native *K. striata* were changed when *K. striata* was grown in the soil conditioned by *S. canadensis* under both monoculture (F_1,6_ = 56.50, P = 0.000 and F_1,6_ = 590.79, P = 0.000 for *G. mosseae* and *G. geosporum* respectively) and mixture (F_1,6_ = 110.52, P = 0.000 and F_1,6_ = 84.98, P = 0.001 for *G. mosseae* and *G. geosporum* respectively). The *S. canadensis*-altered AM fungal community (SC-A-AMF) treatment reduced the relative abundance of DNA of *G. mosseae* but increased that of *G. geosporum* in roots of *K. striata* compared to the initial AMF community (I-AMF) treatment with both monoculture and mixed plantings (Fig. 2).

Culture types (monoculture and mixture) did not affect the relative DNA abundance of *G. mosseae* in roots of native *K. striata* grown in treatments of I-AMF (F_1,6_ = 0.58, P = 0.391) and SC-A-AMF (F_1,6_ = 2.12, P = 0.196) (Fig. 2A). For *G. geosporum*, culture types did not change the relative abundance of DNA in roots of *K. striata* under treatment of I-AMF (_F_1,6_ = 1.92, P = 0.238), but mixture reduced the relative abundance of DNA in roots of *K. striata* under the treatment of SC-A-AMF compared to monoculture (F_1,6_ = 250.89, P = 0.000) (Fig. 2B).

### Interaction between invasive and native plants under the changed AMF community (experiment 2)

**Figure 3:**
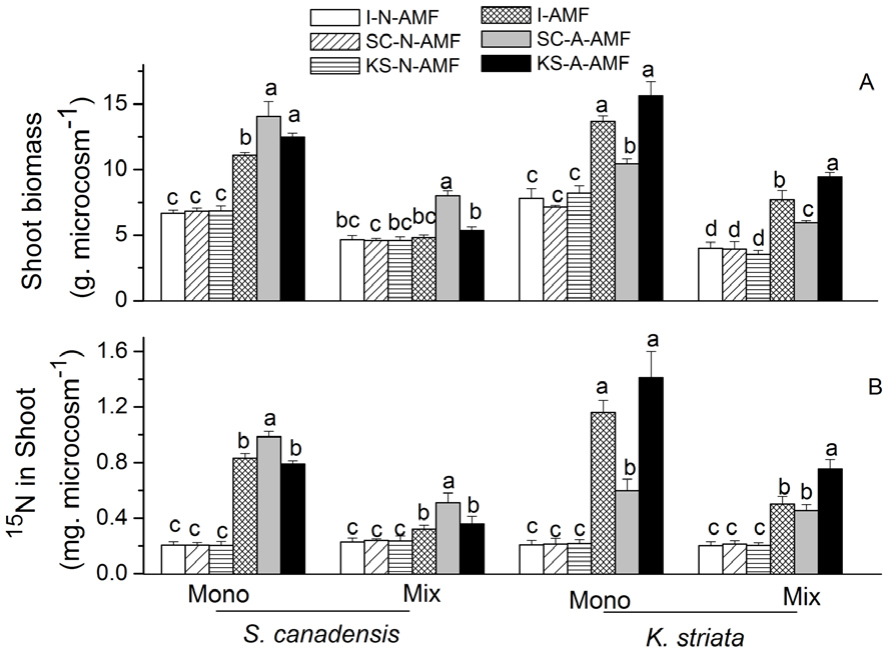
Shoot biomass (A) and shoot ^15^N (B) of invasive and native plants under various AMF communities in experiment 2. I-N-AMF: the initial non-AMF control; SC-N-AMF: the *S. canadensis*-altered non-AMF control; KS-N-AF: the *K. striata*-altered non-AMF control; I-AMF: the initial AMF community; SC-A-AMF: the AMF community altered by the invasive *S. canadensis*; KS-A-AMF: the AMF community altered by the native *K. striata*. Values are means ± standard error. Within each set of six AMF treatments (within monoculture or mixture for each host plant), means with different letters are significantly different at the 5% level.

AMF treatments significantly affected shoot biomass (F_5,18_ = 49.23, P = 0.000 and F_5, 18_ = 33.86, P = 0.000 for *S. canadensis* in monoculture and mixture, respectively; F_5, 18_ = 49.45, P = 0.000 and F_5,18_ = 28.25, P = 0.000 for *K. striata* in monoculture and mixture, respectively). AMF treatments also significantly affected shoot ^15^N (F_5, 18_ = 162.76, P = 0.000 and F_5,23_ = 6.62, P = 0.001 for *S. canadensis* in monoculture and mixture, respectively; F_5,18_ = 31.81, P = 0.001 and F_5,18_ = 27.19, P = 0.006 for *K. striata* in monoculture and mixture, respectively). No significant differences in both shoot biomass and shoot ^15^N were found among the three no-AMF controls for both hosts under monoculture or mixture (Fig. 3, P>0.05). Compared to the I-AMF treatment, the SC-A-AMF treatment enhanced (P<0.05) but the *K. striata*-altered AM fungal community (KS-A-AMF) treatment did not change (P>0.05) the shoot biomass (Fig. 3A) and shoot ^15^N (Fig. 3B) of *S. canadensis*. For *K. striata*, however, shoot biomass (Fig. 3A) and shoot ^15^N (Fig. 3B) decreased (P<0.05) under the SC-A-AMF treatment but increased (P<0.05) under the KS-A-AMF treatment relative to the I-AMF treatment.

**Figure 4:**
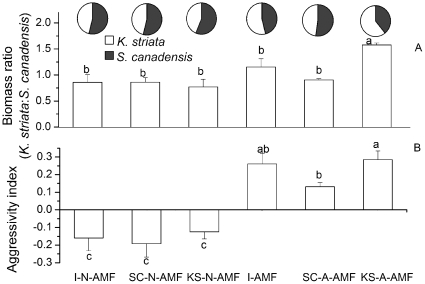
Biomass ratio of *K. striata* to *S. canadensis* (A) and aggressivity index of competition between *K. striata* and *S. canadensis* (B) in response to various AMF communities in experiment 2. The ratio of biomasses and aggressivity index were calculated based on shoot biomass. I-N-AMF: the initial non-AMF control; SC-N-AMF: the *S. canadensis*-altered non-AMF control; KS-N-AF: the *K. striata*-altered non-AMF soil control; I-AMF: the initial AMF community; SC-A-AMF: the AMF community altered by the invasive *S. canadensis*; KS-A-AMF: the AMF community altered by the native *K. striata*. Values are means ± standard error. Means with different letters are significantly different at the 5% level. Inset pie charts represent biomass composition of the two plant species.

AMF communities significantly affected the ratio of *K. striata* to *S. canadensis* biomass in mixture (F_5,18_ = 7.97, P = 0.000). There was no significant difference in biomass ratio among the three non-AMF controls (Fig. 4A, P<0.05). The biomass ratio of *K. striata* to *S. canadensis* was reduced by SC-A-AMF treatment (P<0.05), but was enhanced by the KS-A-AMF treatment (P<0.05) (Fig. 4A).

AMF communities also significantly affected the aggressivity index in the *K. striata*–*S. canadensis* competition (F_5,18_ = 14.67, P = 0.000). No significant difference in aggressivity index was found among the three non-AMF controls (Fig. 4B, P<0.05). Aggressivity index was enhanced by the KS-A-AMF treatment (P>0.05), but was reduced by the SC-A-AMF treatment (P<0.05) (Fig. 4B) relative to the I-AMF treatment.

### Host preference of AMF (experiment 3)

**Figure 5:**
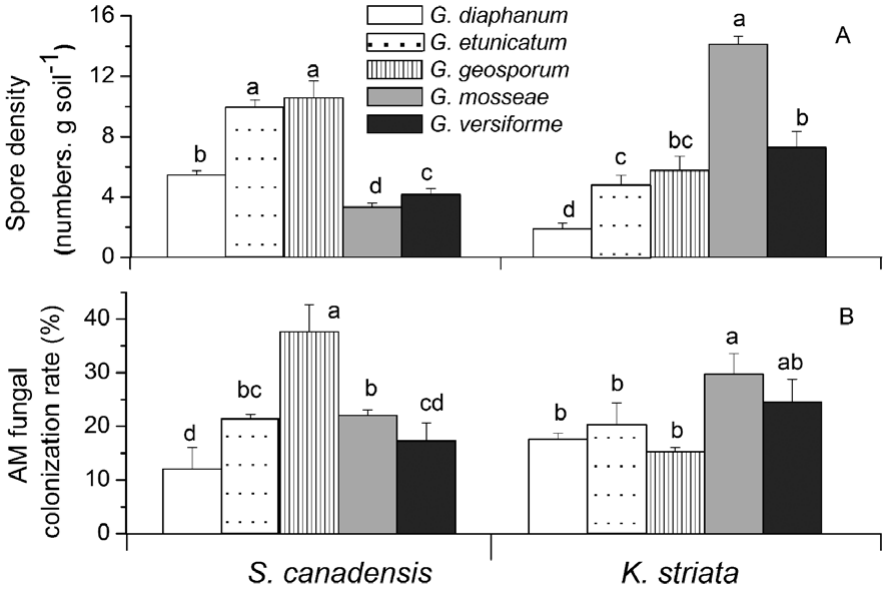
Spore production (A) and root colonization (B) by the five AMF species with *K. striata* or *S. canadensis* as hosts in experiment 3. Values are means ± standard error. For each host plant, means with different letters are significantly different at the 5% level. In the non-AMF treatment, spore numbers and colonization rate were zero (data not shown).

No AMF spores or other indications of colonization were found in non-AMF controls in both host plants. Differences in host preference in the AMF population growth rates were detected in experiment 3 (F_4, 15_ = 36.46, P = 0.000 and F_4, 15_ = 23.91, P = 0.000 for spore density of *S. canadensis* and *K. striata*, respectively; F_4, 15_ = 3.09, P = 0.049 and F_4, 15_ = 3.87, P = 0.023 for colonization rates of *S. canadensis* and *K. striata*, respectively). For *G. geosporum*, spore numbers (Fig. 5A, P<0.05) and colonization rates (Fig. 5B, P<0.05) were higher with *S. canadensis* than with *K. striata*. For *G. mosseae*, however, spore number (Fig. 5A, P<0.05) and colonization rates (Fig. 5B, P<0.05) were higher with *K. striata* than with *S. canadensis*.

**Figure 6:**
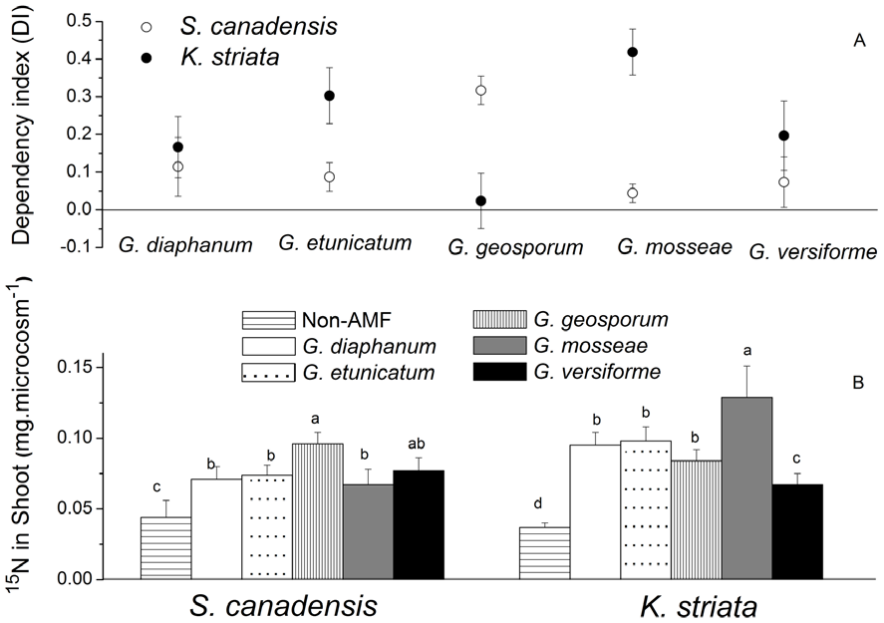
Mycorrhizal dependency index (A) for *K. striata* or *S. canadensis*, and mycorrhizally enhanced ^15^N in shoot biomass (B) as affected by the five AMF species in experiment 3. Note that the ^15^N was added to the compartment without roots and presumably entered the plant via AMF hyphae. Mycorrhizal dependency index = (B_AMF_−B_non-AMF_)/B_AMF_, where B_AMF_ is biomass of the plants in mycorrhizal inoculation treatment and B_non-AMF_ is biomass of the control plants. Values are means ± standard error. For each host plant in (**B**), means with different letters are significantly different at the 5% level.

Plant growth responses (in terms of the dependency index and shoot ^15^N) of the two host plants to AMF inoculation differed among the five AMF species (F_4, 15_ = 14.07, P = 0.000 and F_4, 15_ = 4.65, P = 0.012 for dependency indices of *S. canadensis* and *K. striata*, respectively; F_5, 18_ = 3.12, P = 0.034 and F_5, 18_ = 3.95, P = 0.014 for shoot ^15^N of *S. canadensis* and *K. striata*, respectively). For *S. canadensis*, the dependency index and shoot ^15^N were highest with *G. geosporum* (Fig. 6A and 6B, P<0.05 in both cases). For *K. striata*, the dependency index and shoot ^15^N were highest with *G. mosseae* (Fig. 6A and B, P<0.05 in both cases).

## Discussion

Here we demonstrate that positive feedback between specific mycorrhizal fungi and the invasive plant (*Solidago canadensis*) promoted the invasion success of the invader. The invader altered the spore composition of the AMF communities in that AMF species that were most beneficial for its own growth were promoted at the expense of AMF species that were most beneficial to the native plant species (*Kummerowia striata*).

In our experiment 3, all five AMF species were capable of infecting both *S. canadensis* and *K. striata*. But AMF spore density in soil and hyphal colonization of roots (Fig. 5), both of which reflect AMF population growth [2], indicated that the five AMF species responded differently to the two host plants. *S. canadensis* promoted *G. geosporum* while *K. striata* promoted *G. mosseae*. The dependency index [29] also indicated a high dependency of *S. canadensis* on *G. geosporum* and a high dependency of *K. striata* on *G. mosseae* (Fig. 6A).

We found that the mutualism formed between the AMF species *G. mosseae* and the native plant *K. striata* decreased when this native plant grew in soil in which the AMF spore community had been changed by invasive *S. canadensis* (Fig. 2). Prior experiments have shown that invasive plants degraded AMF mutualisms of native hosts because most invasive species are not mycorrhizal hosts or are less dependent than the native hosts on the mutualism and therefore invest less carbon in maintaining the AMF community [12], [16], [23], [30]. Also, some invasive species can inhibit native mutualisms through allelopathy [31]. In our study, however, the invasive *S. canadensis* is a highly mycorrhizal host [27], and it did not decrease the total abundance of AMF spores in soil after two growing seasons (Fig. 1A). Studies also showed that plant neighbors were important in structuring AMF communities [32] in roots and that the presence of invasive plants changed AMF assemblages in roots of their native neighbors [15]. However, when coexisting with native plants in the same AMF communities, this invasive *S. canadensis* did not change the abundance of *G. mosseae* in native roots (Fig. 2). Based on these results, we suggest that the decrease in mutualism between *G. mosseae* and native plants was due to the decrease of spore density of *G. mosseae* in the AMF community that had been changed by *S. canadensis*.

The degradation of the mutualism between AMF *G. mosseae* and native plants resulted in reductions in nutrient uptake and growth of native plants in our study. The presence of shoot ^15^N indicates that AMF can absorb and deliver N to plants through hyphae because the ^15^N was added to the microcosm compartment that excluded roots but did not exclude AMF hyphae [33]. Our data on shoot ^15^N demonstrated that N uptake by AMF-colonized *K. striata* was reduced when the AMF community had been altered by *S. canadensis* (Fig. 3B). Moreover, host-fungal preference detected in experiment 3 further demonstrated that this reduction was due to a decrease in the abundance of *G. mosseae*, which was the most effective AMF species for *K. striata* (Fig. 6). It is well documented that invasion by non-mycorrhizal or less mycorrhizal species can reduce AMF abundance and disrupt mutualism of native plants [16], [23], [30]. By using ^15^N in the current study, we have expanded this understanding by demonstrating that an invasive plant can reduce the nutrient-acquiring functions of mutualisms by degrading the preferred mutualisms of native plants.

AMF can mediate competition between some invasive and native plants by differently affecting growth of hosts or by transferring carbon between hosts via a shared mycorrhizal network [34]–[37]. The identity of AMF species influencing the performance of invasive plants [11] suggests that the species composition of AMF communities is also important in this mediation. We found that under the *S. canadensis*-changed community in which *G. geosporum* became dominant, the competitive ability of *K. striata* (as indicated by the biomass ratio of *K. striata* to *S. canadensis* and by the aggressivity index [38] was reduced but that of *S. canadensis* was enhanced (Fig. 4). These results suggest that, by shifting the AMF community, the invasive *S. canadensis* generates two kinds of positive feedback that increased its own competiveness: it increased those AMF species that favored its own growth while it decreased the AMF species that favored the growth of its native competitor. Bever [39] found that negative feedback through changes in the composition of the AM fungal community inhibited the dominant plant species leading to the coexistence of the competing plant species. Here, we indicated that the positive feedback through changes in the composition of the AM fungal community promoted the invasive *S. canadensis*, leading to the dominance of the invasive plant and the decline of the native *K. striata*.

One may argue that it is difficult to measure how changes in AMF affect or produce feedback on the interaction between invasive and native plants because of the confounding effects of abiotic factors (i.e., soil nutrients; [40]) and biotic factors (i.e., allelopathy and soil pathogens [17], [41], [42]). The invasive plant used in our study, *S. canadensis*, does exude allelochemicals that interfere with neighboring plants [43] and soil pathogens [44]. We designed non-AMF control treatments (I-N-AMF, SC-N-AMF and KS-N-AMF) corresponding to AMF treatments (I-AMF, SC-A-AMF and KS-A-AMF) during the whole study. We thus can separate allelopathy and other effects from mycorrhizal effect by comparing the biomass ratio and aggressivity index of *K. striata* to *S. canadensis* in each AMF treatment to its corresponding non-AMF control.

Our work increases the understanding of the ecological mechanisms underlying how AMF interactions can participate in feedback affecting plant invasion. Invasive plants may encounter certain novel AMF that facilitate the establishment of the invasive plants [45] or the invasive plants may disrupt AMF mutualisms so as to inhibit native species [16], [23]. Our results indicate that, under greenhouse conditions, an invasive species (*S. canadensis* in our study) can change the dominant species in the AMF community as a consequence of host-AMF preference. This shift in the AMF community generates positive feedback to the invasive plant while reducing preferred mutualisms and competitiveness of native plants (*K. striata* in our study), thus modifying the outcome of the competition in favor of the invasive plant.

## Materials and Methods

### Plants, soil, and AMF species

We used the invasive forb *S. canadensis* L. and the native legume forb *Kummerowia striata* (Thunb.) Schindl. as model plants, and we used soil conditions that matched those of an abandoned agriculture field (about 15 ha) in Zhejiang, China (29°8′N, 121°5′E), where *S. canadensis* has been invasive for 3 years [28], [44]. *Kummerowia striata* is a common weed in crop fields, orchards, and abandoned land [46]. Both *S. canadensis* and *K. striata* are highly dependent on AMF in low nutrient soil [27], [47]. The propagules (ungerminated buds from rhizomes) of *S. canadensis* and seeds of *K. striata* were collected from the abandoned agricultural field. Before the *S. canadensis* invasion, native *K. striata* was the dominant species in this abandoned field.

Five common AMF species (*Glomus mosseae*, *Glomus versiforme*, *Glomus diaphanum*, *Glomus geosporum*, and *Glomus etunicatum*) were selected. These species naturally exist in the abandoned field [28] where the propagules of *S. canadensis* and seeds of *K. striata* were collected. A culture of each of the five AMF was established from a single spore. Cultures were propagated on a common host (*Zea mays* L.) that was grown in sterilized sand (0.45 to 1 mm dia.) in a growth chamber for 4.5 months until sporulation. Each of these AMF has been deposited in Glomales Germplasm Bank in China (Institute of Plant Nutrient & Resources, Beijing Academy of Agriculture & Forestry Sciences). Equal numbers of spores incorporated in soil from five pure cultures were then mixed to create the initial AMF communities (I-AMF) used for the experiments. The soil with spores was used as inoculum.

The surface soil (0–15 cm depth) used in the experiments was obtained from the same abandoned field where the propagules of *S. canadensis* and seeds of *K. striata* were collected. The soil is a sandy loam with a pH of 6.62 (2.5∶1, KCl aqueous solution: soil), 42.14 g kg^−1^ organic matter, and 38.07, 22.99, and 98.23 mg kg^−1^ soil of extractable N (NH_4_-N and NO_3_-N, [48]), extractable P [49] and extractable K (extracted by 2 M HNO_3_ and determined by flame atomic absorption spectrophotometry, flame-AAS), respectively. The soil was air dried, passed through a 5-mm-mesh sieve, and uniformly moistened to constant water content. Then, the prepared soil was mixed with sand (1∶1 by weight) and sterilized by gamma (γ)-radiation.

### Experiment 1

In this experiment, *S. canadensis* and *K. striata* were grown separately in soil containing the mixed initial AMF community (I-AMF). After two growing seasons, the effect of host plant on the AMF community composition was evaluated. The experiment had two plant species (*S. canadensis* and *K. striata*), two AMF treatments (non-AMF [N-AMF] and I-AMF), and four replicates. The N-AMF treatment was not analyzed in experiment 1 but the soil was used as a non-mycorrhizal control in experiment 2.

Each rectangular mesocosm (45 cm long×30 cm wide×20 cm high) with a volume of 27 L was filled with 16 kg of the sterilized loam-sand mixture described above. The soil in half of the mesocosms was inoculated with 500 g of soil containing I-AMF inoculum. The remaining mesocosms received 500 ml of filtrate from 500 g of inoculum (with no mycorrhizal spores) and 500 g sterilized inoculum to correct for possible differences between the microbial communities in mycorrhizal and non-mycorrhizal treatments. Eight propagules of *S. canadensis* or eight seeds of *K. striata* were planted in each mesocosm. Mesocosms were arranged in a greenhouse in a completely randomized block design. Plants were maintained with ambient light and temperature and with air temperature ranging from 18 to 30°C. Plants were watered daily to keep soil moisture at 70–90% of water-holding capacity. No additional nutrients were added.

All the plants were harvested when they naturally senesced after an 8-month growing season (from March to November). Five soil samples (each 100 g) were collected from each mesocosm for monitoring AMF spore density and composition of AMF communities. All of the mesocosms with the remaining soils were stored at 4°C until the following March, when the mesocosms were returned to the greenhouse and planted with the same host as in the first growing season. At the end of the second growing season, soil was again sampled for monitoring density and composition of AMF communities.

Spores were separated from the soil by the wet-sieving method [50]. Spores were counted and identified to species according to the taxonomic information provided by the Glomales Germplasm Bank in China and the VAM website (http://invam.caf.wvu.edu).

To estimate the similarity of AMF communities between the two host plants at the end of each growing season, we subjected the data to analysis by the program PAST (Version, 1.94) [51] and calculated Bray-Curtis similarity.

The data for total spore density of AMF community and spore density of each AMF species under each host plant were first subjected to a homogeneity test and then to a multivariate analysis of variance (MANOVA) (plant hosts as factor and growing seasons as block) using SPSS V.17.0. Treatments were compared by the LSD at the 5% significance level. Bray-Curtis similarity was analyzed with a one-way ANOVA using SPSS V.17.0.

### Experiment 2

Experiment 2 examined how the host-induced alteration in mycorrhizal communities affected the host plants when grown separately or together. Soil samples containing AMF communities under *S. canadensis* (SC-A-AMF) and *K. striata* (KS-A-AMF) from the end of experiment 1 were used as inocula. One kg of soil from each mesocosm at the end of the second growing season in experiment 1 was collected and passed through a sterilized 2-mm sieve to mix the inoculum. The inoculum from each original replication in experiment 1 was used for one replication of experiment 2.

To separate the effects of allelopathy, nutrients, and other rhizosphere factors induced by host plants from the effects of AMF communities, the soils from N-AMF controls under *S. canadensis* (SC-N-AMF) and *K. striata* (KS-N-AMF) in experiment 1 were used as no-AMF inoculum controls corresponding to SC-A-AMF and K.S-A-AMF in experiment 2. Overall, experiment 2 had three kinds of AMF communities and their corresponding non-AMF controls, three kinds of host plants (*S. canadensis*, *K. striata*, and their mixture), and four replicates. The AMF communities and their corresponding non-AMF controls were: the initial non-AMF control (I-N-AMF) and the initial AMF community (I-AMF); the *S. canadensis*-altered non-AMF control soil (SC-N-AMF) and the *S. canadensis*-altered AM fungal community (SC-A-AMF); and the *K. striata*-altered non-AMF soil control (KS-N-AM) and the *K. striata*-altered AM fungal community (KS-A-AMF).

**Figure 7:**
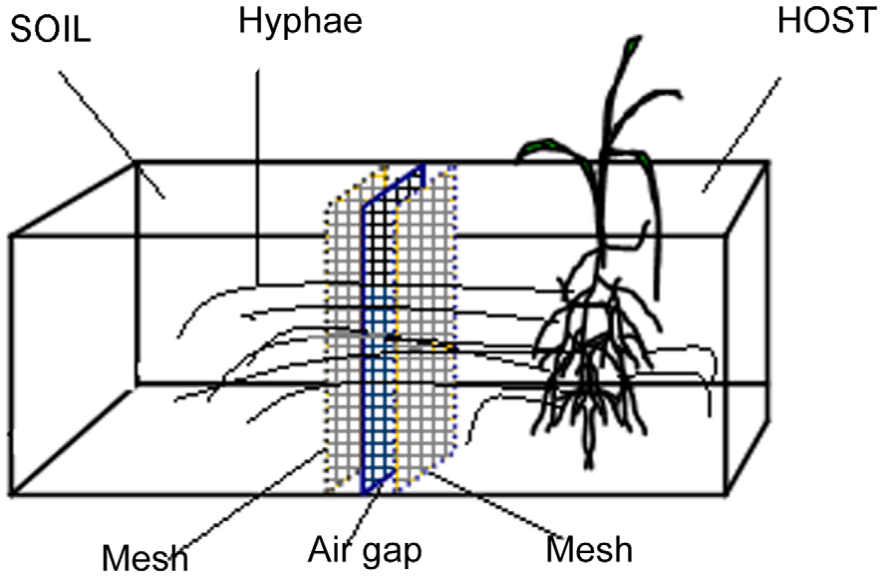
Diagram of a microcosm used in experiments 2 and 3. Each microcosm had two equal-sized compartments, termed the HOST compartment and the SOIL compartment. The compartments were separated by two pieces of nylon mesh with 20-µm openings. A stainless wire net (1.5 mm thick and with 6-mm openings) was inserted between the two pieces of mesh to create an air gap that prevented the diffusion of mobile nutrients between the compartments.

A microcosm containing two compartments (Fig. 7) was designed to assess mycorrhizal contribution to nutrient uptake. Each compartment was 20 cm long×15 cm wide×20 cm high, and the two compartments were separated by two pieces of replaceable nylon mesh (20-µm openings, Tetko/Sefar mesh, Sefar America, New York). To prevent the diffusion of mobile nutrients between the compartments, a stainless wire net (1.5 mm thick and with 6-mm openings) was inserted between the two pieces of replaceable mesh to create an air gap (Fig. 7, modified from Tanaka & Yano [33]). As described in the next paragraph, the compartment containing a plant and AMF was called the HOST compartment, and the other was called the SOIL compartment. The mesh permitted AMF hyphae but not roots to penetrate from the HOST to the SOIL compartment to obtain nutrients.

Each compartment was filled with 3 kg of sterilized 1∶1 soil and sand as described for experiment 1. For treatment SC-A-AMF and its corresponding control (SC-N-AMF), and for treatment KS-A-AMF and its corresponding control (KS-N-AMF), 100 g of soil containing AMF inocula from the end of experiment 1 was incorporated into the soil of each HOST compartment. For the I-AMF treatment, the inocula as used in experiment 1 were incorporated into the soil of HOST compartment. For its corresponding no-AMF control (I-N-AMF), microcosms received 100 ml of washing filtrate from 100 g of AMF inoculum (with no mycorrhizal spores) and equal amounts of inoculum sterilized with γ-radiation to correct for possible differences between the microbial communities in I-AMF and I-N-AMF treatments.

Four healthy germinated seeds of *K. striata*, four propagules of *S. canadensis*, or their combination (two plants of each species) were planted in each HOST compartment. The microcosms were placed in a growth chamber in the greenhouse. All growth conditions were the same as in experiment 1.

The ^15^N tracer was introduced to quantify “mycorrhizally mediated plant N uptake” 3 weeks before the experiment was ended. The ^15^N tracer was injected uniformly as ^15^N-enriched mineral N ((NH_4_)_2_SO_4_, 99.7% atom ^15^N) in deionized water at a rate of 3.0 mg N kg^−1^ soil into each SOIL compartment. When plants were harvested (see next paragraph), the N isotope fraction (^14^N or ^15^N) in shoots was determined using a ThermoFinnigan DELTAPlus continuous flow isotope ratio mass spectrometer (CF-IRMS, Thermo Finnigan DELTA Plus, Waltham, MA, USA). Sample ^15^N (%) was converted to excess N isotope (mg) based on the atom ratio of atmospheric N. Sample ^15^N content was then calculated from fractional abundance (^15^N/(^14^N+^15^N)) and total N content [52].

The plants were harvested 6 months after planting when both invasive and native plants were flowering. Root systems were separated from shoots, and the fresh roots were weighed immediately. Half of each root sample was frozen at −80°C for molecular analysis. The remaining half of each sample was used for measurement of dry root biomass. The shoots and roots were dried at 65°C for 48 h and weighed to determine dry shoot and root biomass. Shoot biomass of *S. canadensis* and *K. striata* in mixture were used to calculate the ratio of *K. striata* to *S. canadensis*.

Several methods can be used to quantify the abundance of specific AMF in roots, and these methods include real-time polymerase chain reaction (PCR) [53]–[56]. Here we used a nested PCR-denaturing gradient gel electrophoresis (DGGE)-sequencing method [54]–[56] to measure the relative abundance of DNA of the AMF species *G. mosseae* and *G. geosporum* in the native roots. Two kinds of primers were used. One primer, AM1/NS31 [57], is specific for all AMF species, and the other primer, NS31-GC/Glol [58], is specific for the AMF species in the genus *Glomus*.

Briefly, total DNA of root samples was extracted using a DNA Extraction Kit and following the manufacturer's protocol (Axygen Biosciences). Isolated DNA was subjected to nested PCR with primers AM1/NS31 and NS31-GC/Glol. Thermocycling program and conditions for the first PCR with primers AM1/NS31 were 95°C 5 min; followed by 35 cycles of 95°C 30 sec, 64°C 1 min, and 72°C 2 min; and a final extension at 72°C for 10 min. The 50-µl reaction volume contained 1 µl of dNTP, 1 µl of each primer (10 pmol), 5 µl of 10× buffer, 1 µl of template, 0.5 µl of Taq polymerase, and ddH_2_O. The 550-bp PCR product [57] and primer specificity were analyzed by agarose gel electrophoresis (1.0% (w/v) agarose, 100V, 60min) and ethidium bromide staining in the presence of the PUC19 DNA marker. The thermocycling program and conditions for the second PCR with primers NS31-GC/Glol were 94°C for 5min; followed by 35 cycles of 94°C 45 sec, 55°C 1min, and 72°C 45 sec; and a final extension at 72°C for 10 min. The PCR reaction was carried out with a Tgradient DNA thermal cycler (Whatman Biometra, Germany). The nested PCR amplicons were first checked by agarose gel electrophoresis (1.7% (w/v) agarose, 100V, 60 min) and ethidium bromide staining to determine size (approximately 270 bp) and yield in the presence of the pBR322 DNA/Alul Marker. Then the nested PCR products were used for DGGE analysis following the procedure described by Muyzer *et al.* [59] and Liang *et al.* [55] and by using a D-Gene system (Bio-Rad Laboratories, Hercules, CA, USA) at a constant temperature of 60°C. Electrophoresis was for 10 min at 200 V, after which the voltage was lowered to 150 V for an additional 7 h. Gels were stained in 1×TAE containing 4 ml Sybr Green per 20 ml TAE, and gel images were digitally captured using the ChemiDoc EQ system. The DGGE band pattern and intensity were analyzed by Quantity One Software (Bio-rad, Hercules, CA, USA). To obtain sequences from DGGE bands, each band in DGGE was excised. Then the DNA in the band was eluted and reamplified with primer Glo1/NS31 (no GC-clamp added) following the PCR procedure described above. The reamplified PCR products were sequenced by the Shanghai Sangon Biological Engineering Technology & Services Co., Ltd. Similarity comparison of each DNA sequence recovered from the DGGE gel was performed using an online program (BLAST, http://www.ncbi.nlm.gov/BLAST). The specific bands for *G. mosseae* and *G. geosporum* in the DGGE were identified through this sequence similarity comparison. The sequences of *G. mosseae* and *G. geosporum* were submitted to GenBank database for verification. The accession numbers are GU978970 for *G. mosseae*s and HM853685 for *G. geosporum*.

The total intensity of all bands and the bands representing *G. mosseae* and *G. geosporum* in the same profile were used to calculate the relative abundances of DNA *G. mosseae* and *G. geosporum*. The relative abundance DNA of *G. mosseae* or *G. geosporum* (%) = I_g_/I_t_×100, where I_g_ is the intensity of the *G. mosseae* band or *G. geosporum* band, and I_t_ is the total intensity of all the AMF species bands in one profile.

The aggressivity indices of plants [38] were calculated using shoot biomass of *K. striata* and *S. canadensis* in monoculture and mixture. Aggressivity index = (Y_ij_/Y_ii_)−(Y_ji_/Y_jj_), where Y_ij_ and Y_ii_ are the shoot biomass of *K. striata* in monoculture and mixture, and Y_ji_ and Y_jj_ are the shoot biomass of *S. canadensis* in monoculture and mixture.

Differences in shoot biomass and shoot ^15^N between AMF treatments for each host plant in monoculture and mixture were separately analyzed (one analysis for monoculture and one for mixture) with a one-way ANOVA using the general linear model procedure in SPSS (V.17.0). Differences in shoot biomass ratio (*K. striata*: *S. canadensis*) and aggressivity indices in the competition between *K. striata* and *S. canadensis*, as affected by AMF community, were also analyzed with a one-way ANOVA. The relative abundance of DNA of *G. mosseae* or *G. geosporum* in roots of *K. striata*, as affected by AMF community or plant culture types, was analyzed separately with a one-way ANOVA. The relative abundance of DNA of AMF species, biomass ratios, and aggressivity indices were arcsine transformed to satisfy variance assumptions before ANOVAs were performed. When ANOVAs were significant, means were compared by least significant difference (LSD) at the 5% significance level.

### Experiment 3

Experiment 3 determined whether any of the five AMF species preferred the invasive host to the native host or vice versa. There were five AMF species and a non-AMF control, two plant species, and four replications. AMF treatments received 100 g of soil containing AMF inocula. The non-AMF control received equal amounts of inoculum sterilized with γ-radiation plus non-AMF filtrate from the inoculum, thereby controlling for potential mineral and non-mycorrhizal microbial components of the inoculum. *S. canadensis* or *K. striata* were grown in the HOST compartment of the microcosms described for experiment 2. Three kg of sterile soil and sand mix (1∶1 w/w) plus the AMF inoculum was added to each compartment. The ^15^N tracer was introduced to the SOIL compartment of the microcosms as described for experiment 2.

The plants were grown under the same conditions as described for experiments 1 and 2. Six months after planting, the plants were harvested. Root systems were separated from shoots, and the fresh roots were weighed immediately. Half of each root sample was used for quantification of AMF colonization (see next paragraph). The remaining half of each sample was oven-dried (65°C for 48 h) and used for measurement of dry root biomass. Measurements for plant biomass and ^15^N in shoots were the same as described for experiment 2.

AMF colonization of roots was quantified using a microscope (×20 magnification) and the gridline intersection method developed by Giovannetti & Mosse [60]; 200 transects were examined per replicate. Measurement of spore density was the same as described for experiment 1. The mycorrhizal dependency index (DI) of host plants for each AMF species [29] was calculated using biomass of *S. canadensis* and *K. striata* in the AMF inoculation treatments and the non-AMF control. DI = (B_AMF_−B_non-AMF_)/B_AMF_, where B_AMF_ is biomass of the plants in the mycorrhizal inoculation treatment and B_non-AMF_ is biomass of the control plants.

For each host plant species, one-way ANOVAs (with AMF species as the factor) were performed on the dependent variables of shoot N ^15^ and DI. In the non-AMF treatments, spore numbers and colonization rate were always zero, and this treatment was not included when one-way ANOVAs were used to compare spore numbers and colonization rates between AMF treatments. Treatments were compared using LSD at the 5% significance level. Data for AMF colonization rate and DI were arcsine transformed before ANOVAs were performed.
